# Do Social Casino Gamers Migrate to Online Gambling? An Assessment of Migration Rate and Potential Predictors

**DOI:** 10.1007/s10899-014-9511-0

**Published:** 2014-11-20

**Authors:** Hyoun S. Kim, Michael J. A. Wohl, Melissa M. Salmon, Rina Gupta, Jeffrey Derevensky

**Affiliations:** 1 Department of Psychology, Carleton University, 1125 Colonel By Drive, B550 Loeb Building, Ottawa, ON K1S 5B6 Canada; 2 McGill University, Montreal, QC Canada

**Keywords:** Social casino, Online gambling, Gambling, Migration, Micro-transactions, Time, Gambling motivations, Enhancement, Skill building, Credits

## Abstract

Social casino games (i.e., free-to-play online gambling games) are enjoyed by millions of players worldwide on a daily basis. Despite being free to play, social casino games share many similarities to traditional casino games. As such, concerns have been raised as to whether social casino games influences the migration to online gambling among people who have not engaged in such activity (see Griffiths in World Online Gambl 9:12–13, 2010). To date, however, no empirical research has assessed this possibility. Thus, the purpose of the present research was to assess the extent to which social casino gamers migrate to online gambling and potential predictors (time spent on social casino games, skill building, enhancement and micro-transactions) of such migration. To this end, social casino gamers who never gambled online (N = 409) completed a questionnaire battery assessing our variables of interest and were re-contacted 6-months later to see if they had engaged in online gambling during the intervening months. Approximately 26 % of social casino gamers reported having migrated to online gambling. Importantly, engagement in micro-transactions was the only unique predictor of migration from social casino gaming to online gambling. The implications for the potential harms associated with social casino gaming are discussed.

## Introduction

Social casino games (i.e., free-to-play online gambling games) are immensely popular and generate a handsome profit for providers. Indeed, there were over 100 million active players of social casino games (Takahashi 2012a) who generated $1.6 billion (USD) in revenue between 2011 and 2012 (Takahashi 2012b). According to the Morgan Stanley Report (2012), social casino gaming is expected to increase at a rate of 100 % per year with projected revenues of $2.6 billion (USD) by 2015. These are astonishing numbers considering that unlike traditional casino games, social casino games do not involve the wagering of money and outcomes do not yield prizes with monetary value. With that said, the structural characteristics of social casino games mimic those of traditional casino games (Gainsbury et al. 2014a). In this way, social casino games may serve as a training ground for gambling (see Griffiths 2010, 2013; Parke et al. 2013).

At present, however, there is a paucity of empirical research on the consequences of playing social casino games. What is known is that people who engage in social casino gaming are more likely to engage in online gambling compared to people who do not play social casino games (Gainsbury et al. 2014b; Parke et al. 2013). Unknown is whether social casino gaming motivates a migration to online gambling among people who have not previously engaged in such activity. Herein, we address this empirical gap. Specifically, we empirically assessed whether social casino gamers who have never gambled online migrate to online gambling over a 6-month span (i.e., change rate). More importantly, we also examined possible predictors of migration, such as, motivation to play social casino games (skill building, enhancement), perceived time spent playing social casino games, and the purchase of virtual credits on social casino games (known as micro-transactions).

### Social Casino Games: By the Numbers

According to Gainsbury et al. (2014a) social casino games (a) do not require monetary payment to play, (b) are based on or have substantial interaction with a social media platform, and (c) are themed to simulate gambling activities. Rather than requiring payment or wagering monetary value, social casino games operate on a *freemium* model in which players are provided free credits that are reloaded periodically for the player to use. Moreover, players are given the opportunity and are actively encouraged to purchase additional credits or virtual items (i.e., micro-transactions) from the gaming operator in order to continue play or engage in higher stake bets.

Social casino games are offered through a variety of social networking sites, the most popular of which is Facebook (Gainsbury et al. 2014a; Zainzinger 2012). Social networking sites offer the ideal launch pad for social casino games, in part, due to the high number of active users. Currently, one in four (approximately 1.47 billion) people around the globe are users of social networking sites and this number is expected to grow to 2.6 billion users by 2018 (eMarketer 2013). Although social networking sites were created with the intention of helping friends and family stay in contact, they have become the hub of gaming activities via applications that can be added to a person’s profile. Importantly for the current research, social casino games are among the top rated games available on social networking sites (Takahashi 2013).

Indeed, Double Down Casino reported having over 6 million monthly active users in 2012 (Stradbrooke 2013) and Party Poker boasted having approximately 26 million monthly active users (Jones 2012). In total, there are an estimated 170 million people engaging in social casino games on a monthly basis, which is more than triple the number of people engaging in online gambling (Morgan Stanley Report 2012).

### Convergence of Social Casino Games and Online Gambling

According to the Morgan Stanley Report (2012), the vast population of social casino gamers represents a potential pool of online gamblers that may be recruited to increase revenues. In this spirit, gambling operators have begun purchasing, merging, and partnering with social casino gaming companies (Sapsted 2013; Schneider 2012). As evidence, International Game Technology (IGT; a Nevada-based company that designs and manufactures slot machines) recently purchased Double Down Casino (a social casino gaming company) for $500 million in 2012 (PRNewswire 2012) and Zynga (a popular supplier of social casino games) has merged with Bwin Interactive (an online gambling operator). The ongoing alignment between social casino games and online gambling are bound to blur the lines between the two products (see Gainsbury et al. 2014a). Indeed, anecdotal evidence from social casino gamers suggested that it becomes increasingly difficult for them to distinguish when they are playing social casino games and when they are playing online gambling games (Parke et al. 2013).

To facilitate the alignment between social casino gaming and online gambling, online gambling operators often place target advertisements for their sites on the Facebook page of social casino gamers, in addition to embedding these advertisements in the social casino game itself (see Parke et al. 2013). Their explicit hope is that social casino gamers will decide to move from playing for *fun money* to playing for *real money* (Morgan Stanley Report 2012). As a result, there have been calls to assess the potential impact that social casino games have on peoples gambling behavior (Gainsbury et al. 2014a; King et al. 2010; Parke et al. 2013).

To date, however, no study has directly tested whether social casino games act as a conduit for online gambling. However, there is research suggestive of such a link. For example, Gainsbury et al. (2014b) found that social casino gamers share many similar socio-demographic characteristics (e.g., employment, education, income) with online gamblers. Given these similarities, it is perhaps not surprising that a strong predictor of online gambling is engagement in social casino games (King et al. 2012). Putting a dark line under these findings, over half (58.3 %) of disordered gamblers who were seeking treatment stated that social casino games were their first experiences with gambling (Parke et al. 2013).

Due to the correlational nature of the aforementioned research, whether social casino games *lead* people to online gambling is not known. Online gamblers may simply be attracted to and engage in social casino gaming rather than social casino gaming influencing their migration to online gambling. Needed, therefore, is a longitudinal assessment of people who play social casino games, but have yet to gamble online. Such a methodological approach would help determine a rate of migration (if it occurs) as well as potential predictors of migration. To this end, the purpose of the present research was to determine whether (a) social casino gaming influences migration to online gambling (i.e., migration rate) and (b) to identify potential predictors of the migration to online gambling.

### Migrating from Play for Fun to Play for Pay

Herein, we focused our attention on the following potential predictors of migration from social casino gaming to online gambling: perceived time spent playing social casino games, desire to skill build, self-enhancement motivations, and whether participants purchased virtual credits (i.e., micro-transactions). These potential predictors of migration were chosen based on a review of the literature (including grey literature) presented herein, as well as, the outcome of focus groups that the authors previously conducted with social casino gamers (Gupta et al. 2013). It is understood that the potential predictors assessed in the current study are not exhaustive. Moreover, our choice of potential predictors should not be taken as a value judgment on whether variables in other domains (e.g., socio-demographics) are of lesser import. With that said, we believe the time spent on social casino games, desire to skill build, self-enhancement motivations, and engagement in micro-transactions provide a good starting point in the assessment of possible migration from social casino gaming to online gambling. Below we provide a brief summary of why each potential predictor was chosen for assessment.

#### Time Spent

One of the greatest theorized harms of social casino gaming is the normalization of gambling behaviors (Griffiths 2010; Parke et al. 2013). The more time a person spends playing social casino games, the more likely it is for the person to see gambling as an acceptable everyday activity and may develop positive attitudes toward gambling. Indeed, social casino gamers were more likely than non-social casino gamblers to state that the benefits of gambling outweigh the harms (Gainsbury et al. 2014b). Thus, it was hypothesized that the more time a player spends engaged in social casino gaming, the greater the likelihood of migrating to online gambling.

#### Skill Building

In a qualitative study with online gamblers and social media users, Gupta et al. (2013) noted many of their participants played social casino games to build and reinforce their skills or practice before making the migration to online gambling. That is, social casino gamers used the free sites as a training ground prior to migrating to paid sites. This is of concern as social casino games offer higher payout rates than their gambling counterparts (Sevigny et al. 2005). For example, Sevigny et al. (2005) found that almost 40 % of social casino gaming sites offered slot machine payout rates greater than 100 %. These inflated payout rates are problematic as past research suggests that higher payout rates early on may lead to the development of problematic gambling behaviors (Harrigan and Dixon 2009) where gamblers persist longer in the face of continued loss (Young et al. 2008). Moreover, King et al. (2010) have argued that engaging in social casino games may misrepresent the true amount of skill involved in gambling through distorting the odds of winning, which may lead to the desire to engage in gambling for real money.

#### Enhancement

Social casino games are designed to increase player experience by maximizing enjoyment and providing a sense of excitement to the social casino gamer (Derevensky et al. 2013; Sapsted 2013). However, the amount of excitement one can experience when wagering nothing of monetary value may be limited. Indeed, to some gamblers, it is the element of wagering money on an uncertain outcome that provides the excitement and adrenaline rush (see Wulfert et al. 2008). Thus, social casino gamers who play social casino games for enhancement motives may need to increase their stakes in order to feel similar levels of excitement. One avenue of doing so is to migrate from playing for fun to playing for pay. Moreover, past research has shown that much like alcohol and other psychoactive substance dependencies, gamblers are prone to developing a tolerance to their habitual gambling activities, and therefore need to increase the size or frequency of their bets to obtain the same level of excitement (Derevensky 2012; Griffiths 1993). As such, social casino gamers may seek to migrate to gambling for real money in order to obtain the same level of excitement previously provided through social casino games.

#### Micro-transactions

According to Griffiths (2013), the purchase of virtual credits or virtual items makes the activity of social casino gaming more similar to gambling. Thus, micro-transactions may be a crucial predictor in the migration to online gambling, as these players have now crossed a line by paying to engage in these activities. Although, only 1–5 % of social casino gamers make micro-transactions (Casual Games Association 2012; Delaney 2014; Parke et al. 2013) those who purchase virtual credits spend an average of $78 (Casual Games Association 2012; Takahashi 2012b). Despite the limited numbers of social casino gamers purchasing virtual credits, revenues from micro-transactions account for 60 % of all social casino gaming revenue (Casual Games Association 2012). Thus, a significant amount of revenue is based on players’ desire to purchase virtual credits above and beyond what is provided to the player in seed credits. However, unlike online gambling, social casino games do not provide an opportunity to win money back, or to win more than one’s initial wager. As such, social casino gamers who make in-game purchases may decide to migrate to online gambling sites where they have a chance (albeit slim) to either win more than their initial wager or win back their initial wager.

## Overview of the Current Research

To assess whether social casino gaming leads some people to migrate to online gambling, a community sample of social casino gamers were recruited for a longitudinal study. Whilst previous researchers (Griffiths 2013; King et al. 2010; Meerkamper 2010) have theorized the harms of social casino gaming among adolescents and youths, recent studies suggest the demographics of social casino gamers are not limited to young adults. For example, according to Casual Games Association (2012), the average age of the social casino gamer is 35 years. Providing further support for this contention, the mean age of social casino gamers in Gainsbury et al.’ (2014b) study was 43 years. Therefore, a community sample of social casino gamers was recruited to assess the migration from social casino gaming to online gambling. It was hypothesized that (a) the more time participants perceived engaging in social casino gaming, (b) the more a person endorsed playing for skill building and enhancement, and (c) whether or not a participant had made a micro-transaction would predict the migration from social casino gaming to online gambling.

## Method

### Participants

Participants consisted of 409 people (212 male, 194 female, 3 unreported) recruited from the United States via Amazon’s Mechanical Turk (MTurk) system. Participation was limited to those who played social casino games and, importantly, who had not previously gambled online for real money. The participants ranged in age from 18 to 69 years (*M* = 30.31, *SD* = 9.62) and were compensated US $.50 for the initial session and US $.25 for completing the 6-month follow-up. Remuneration was based on normative rates for conducting such a study on MTurk (see Mason and Suri 2012). Remuneration tends to be low because people on MTurk participate in research out of interest or to pass the time rather than for the sake of monetary compensation, making MTurk a good source of data (Buhrmester et al. 2011).

Out of the initial 409 participants, 324 (79.23 %) gave permission to be re-contacted, and 100 (30.86 %) participants completed the 6-month follow-up. One participant was excluded from analyses because (s)he did not provide information to link his or her data to the initial session. Thus, the final 6-month follow-up study consisted of 99 participants (43 male, 55 female, 1 unreported), with age ranging from 18 to 69 years (*M* = 33.38, *SD* = 10.56).

### Procedure

Participants who agreed to participate were provided a link to the online survey hosted by Qualtrics. After providing consent, participants completed a series of questionnaires measuring the variables of interest and were directed to the informed consent for re-contact and the process-debriefing pages. Those who granted permission to be re-contacted were contacted 6 months later via email with a link to the follow-up online survey that was once again hosted by Qualtrics. After obtaining informed consent, participants completed an additional series of questionnaires measuring the variables of interest and were re-directed to the final debriefing page.

An Ethics Certificate was obtained to conduct this study from the Research Ethics Board at the authors’ home institution.

### Measures

During the initial session, participants completed a broad-based questionnaire about their social casino play. To assess time spent playing social casino games, participants completed a measure assessing their perceived time spent playing social casino games (e.g., *“I spend a lot of time playing free online gambling games”*). The response was anchored from 1 (*strongly disagree)* to 7 (*strongly* agree). Three items (*α* = .59) were included to assess enhancement motives to engage in social casino games. These items were anchored at 1 (*strongly disagree*) and 7 (*strongly agree*). Examples of items include: *“To relieve boredom,” “Fun, enjoyment, and entertainment,”* and *“Excitement and thrills.”* Two items were included to assess social casino gaming as a means to skill build for the purpose of gambling. These items were: *“To improve skills before playing with real money,”* and *“For the competition*.” Both items were anchored at 1 (*strongly disagree*) and 7 (*strongly agree*). Lastly, participants were asked whether or not they had paid for credits or tokens while playing social casino games. Response options to this item were 1 (*yes*) or 0 (*no*).

In a brief 6-month follow-up session, participants responded to the following item: *“Have you ever gambled online for money?”* Response options to this item were 1 (*yes*) or 0 (*no*).

## Results

### Preliminary Analysis

To ensure no difference existed between participants who completed the 6-month follow-up and those who did not on our predictor variables (perceived time spent, enhancement, skill-building and micro-transactions), a one-way Analysis of Variance (ANOVA) was conducted between the groups. The results suggest no differences exist between the groups on any of our predictor variables at Time 1. Specifically, there were no between group differences on time spent (*p* = .07), enhancement (*p* = .55), skill building (*p* = .53), or micro-transactions (*p* = .95).

### Migration to Online Gambling

#### Migration Rate

Of the 99 participants who completed the 6-month follow-up, 26 (26.26 %) indicated that they had gambled online for real money (17 male, 9 female).^1^^,^ 2 Of the participants who migrated to online gambling, 25 (96.15 %) were Caucasian and one (3.85 %) was South Asian (i.e., Indian). Furthermore, of the participants who migrated to online gambling, two (7.69 %) were unemployed, four (15.39 %) were employed part-time, and 20 (76.92 %) were employed full-time.

#### Predictors of Migrations

To assess which factors predicted migration from social casino gaming to online gambling, a simultaneous binary logistic regression was performed with migration to online gambling as the dependent variable, and the four variables of interest as the predictor variables. The results are summarized in Table 1.

**Table 1:** Summary of binary logistic regression for predictors of migration from social casino gaming to online gambling

Variable	*B*	*SE*	Wald’s χ^2^	*df*	Sig	*Exp*(*B*)	95 CI	
							LL	UL
Intercept	−1.36	.28	24.37	1	<.001	.26		
Time spent	.15	.17	.78	1	.38	1.16	.83	1.62
Skill building	.08	.17	.24	1	.62	1.01	.78	1.52
Enhancement	−.37	.29	1.68	1	.20	.69	.39	1.21
Micro-transactions	2.10	.73	8.23	1	.004	8.16	1.94	34.26

#### Baseline Migration

There was a significant reduction of the odds of migration from social casino gaming to online gambling when all assessed predictors were held at a fixed value, Wald’s χ^2^ (1) = 24.37, *p* < .001, *OR* = .26. Specifically, the chances of migration holding all the predictor variables constant, were only about 26 % of the odds of not migrating.

#### Time Spent

Time spent playing social casino games was not a significant predictor of migration to online gambling, Wald’s χ^2^ (1) = .78, *p* = .38, confidence interval (CI) [.83, 1.62], *OR* = 1.16. Specifically, when holding the other predictors at a fixed value, the odds of migration were unchanged by perceived time playing social casino games.

#### Skill Building

When holding the other predictors at a fixed value, playing social casino games in order to increase skill at the game did not significantly influence the odds of migration to online gambling, Wald’s χ^2^ (1) = .24, *p* = .62, CI[.78, 1.52], *OR* = 1.09.

#### Self-enhancement Motivation

When holding the other predictors at a fixed value, playing social casino games for enhancement in our initial survey did not significantly influence the odds of migration to online gambling, Wald’s χ^2^ (1) = 1.68, *p* = .20, CI[.39, 1.21], *OR* = .69.

#### Micro-transaction

The odds of migration from social casino gaming to online gambling was significantly influenced by micro-transaction engagement (holding the other predictors at a fixed value), Wald’s χ^2^ (1) = 8.23, *p* = .004, CI [1.94, 34.26], *OR* = 8.16. Specifically, the odds of migration to online gambling were approximately eight times greater among people who made micro-transactions on social casino games compared to social casino gamers who did not make micro-transactions, controlling for the other predictors.

## Discussion

Although engagement in social casino games is a daily pastime for millions of people, concerns have been raised about whether they serve as a gateway to online gambling (Griffiths 2010, 2013; Parke et al. 2013). Providing the first empirical support for this contention, the current study found over a quarter of social gamers migrated to online gaming. This rate of migration is alarming considering that there are an estimated 800 million social casino gamers worldwide (Morgan Stanley Report 2012)—a target pool of potential recruits for the gambling industry. Indeed, if the results of the current study are extrapolated to the global constituency of social casino gamers, then the gambling industry might expect a staggering 200 million new online gamblers from the current population of social casino gamers. Moreover, they need not wait long for the migration to take place—participants who migrated did so in less than 6 months.

Importantly, the current research also assessed potential predictors of migration from social casino gaming to online gambling. To this end, motivation to play social casino games (e.g., enhancement and skill building), perceived time spent playing social casino games and whether participants made a micro-transaction (i.e., purchasing of virtual credits) were assessed. The results indicate micro-transactions were the only significant unique predictor of transition from social casino gaming to online gambling. Moreover, the effect of micro-transaction engagement was very strong. Specifically, the odds of migration to online gambling were eight times higher among social casino gamers who purchased virtual credits compared to social casino gamers who did not purchase virtual credits.

Perhaps most surprising was that participants’ expressed motivation to initiate social casino gaming was not a significant predictor of migration to online gambling. Neither playing to build skills nor playing for self-enhancement significantly predicted the migration to online gambling. One possible explanation for this result is that 6 months was not sufficient time for skill building to occur. That is, social casino gamers who play for the purpose of skill building may need longer than 6 months to feel sufficiently skilled to migrate. Additionally, it is possible that some social casino gamers who play to build their skills may subsequently develop insight to the fact that, over time, the gambler is bound to lose, which may decrease their desire to gamble online. In terms of self-enhancement motives, it is possible that because achievements on social casino games are announced to friends who are on social media, the need for self-enhancement is satisfied by the social casino game itself (and thus, there is no need to engage in online gambling).

Lastly, it is possible that *why* people start playing social casino games is less important for migration to online gambling than what occurs whilst playing social casino games. Indeed, the results of the current study suggest that purchasing virtual credits (i.e., micro-transactions) significantly increased the odds of migration (when controlling for the other possible predictors of migration). This result is in line with Griffiths’ (2013) contention that providing social casino gamers with the ability to purchase virtual credits to enhance play was a masterstroke by the gaming industry. This is because purchasing virtual credits to engage in or extend play makes the activity psychologically closer to gambling than a video game. In this way, the social gamer is primed to spend money on gambling-styled games online. Thus, micro-transactions may act as a ‘foot in the door’ for online gambling. Providing additional substance behind such a claim, Gupta et al. (2013) reported one reason that social media users are reluctant to gamble online is a reluctance to provide credit card information online—there is a lack of trust in providers to keep their personal information private. Social casino gamers who make micro-transactions lack (or have overcome) such issues of trust. Once the micro-transaction boundary has been crossed, it may then be easier to do so again with other aspects of the online world.

It is also possible that social casino gamers who have engaged in micro-transactions might rationalize their migration to online gambling by thinking that money spent on online gambling (where there is an opportunity to win money) is better spent than on social casino games (where no money can be won). Given that there is no chance to win money at social casino games, there is more than a touch of logic to such a rationalization, despite the fact that the odds suggest gamblers will lose any money invested in gambling over time (Blanche 1950).

### Implications

The present research has important implications for preventing the possible migration to online gambling. Currently, there are no mandatory governmental regulations for the social casino gaming industry; rather, they have a recommended framework for best practices. Furthermore, there are no age restrictions in practice. The lack of regulation has resulted in many social casino games providing misleading and/or inflated payout rates. Though these inflated payout rates are designed to keep people playing social casino games, this may result in misperceptions about the odds and chances of winning if they migrate to online gambling. Indeed participants in Gupta et al.’ (2013) and Parke et al.’ (2013) research indicated that winning large sums of virtual credits on social casino gaming sites was a key reason for their migration to online gambling.

Ralph Topping, CEO of William Hill, is a proponent of regulation for social casino gaming (cited in Schneider 2012). Topping argued that social casino gaming is already the “next big thing” in the gambling industry and that social casino gaming carries the same risks as online gambling in the development of disordered gambling. However, up until now, there has been no empirical research demonstrating the effects of social casino gaming on future gambling behaviors. The results of the current research provide preliminary support that social casino games should be regulated at least to some degree to provide warning signs in order to minimize the migration to online gambling.

### Limitations

Some limitations from the present study should be noted. While every effort was made to contact social casino gamers who agreed to participate in the follow-up study, the rate of attrition was relatively high. As such, it is possible that those who did not complete the 6-month follow-up psychologically differed from participants who completed the follow-up study. For example, those who did not complete the 6-month follow-up may have been more likely to migrate to online gambling and thus may have felt embarrassed, guilt or shame. If so, there might be a greater reluctance for individuals to participate in follow-up sessions (see Wohl and Sztainert 2011). Having said that, our preliminary results showed that there were no significant differences on the assessed variables at Time 1. Thus, the findings and their generalizations lend some degree of confidence.

Second, while the results of the current study support the notion that social casino gamers do in fact migrate to online gambling, the current study did not assess whether any harms were incurred due to migration to online gambling. It was beyond the scope of this research to assess whether social casino gamers who migrated to online gambling might develop symptoms of disordered gambling. However, a study assessing whether social casino gamers face harm due to the migration to online gambling would prove informative.

Third, we note that there are likely to be other important predictors that were not assessed in the present research that may predict the migration from social casino gaming to online gambling. While the predictors assessed in the current research were based on the literature reviewed herein, there are likely other important variables that influence the migration from social casino gaming to online gambling. For example, dispositional characteristics such as impulsivity, sensation seeking and reward sensitivity may all play a role in the migration to online gambling. Moreover, cognitive distortions as a result of misleading payout rates may also be an important factor that leads social casino gamers to online gambling. Social casino games are a new phenomenon and there is a paucity of empirical research in this domain. Future research on social casino gaming would benefit from studies that assess causal pathways, potential moderators and confounders of the migration to online gambling.

Lastly, it is important to note that the CI range for the significant effect of micro-transactions on migration was rather wide (1.94–32.46). Thus, although a large effect was observed, the true effect might not be as large as it appears to be based on the point estimate. This is because the CI describes the uncertainty inherent in the estimate, and describes a range of values within which we can be reasonably sure that the true effect lies. Importantly, this range can be influenced by sample size—sample size and range are typically inversely related. With a narrow CI, the effect size is known precisely. As the CI widens the uncertainty is greater, although there may still be enough precision to make decisions about the utility of the intervention. In the current study, 26 of the 99 participants migrated to online gambling. As such, we did not have a great deal of statistical power to detect significant effects. When statistical power to detect an effect is low, typically only large effects are detected as significant. Herein, a large micro-transaction effect was observed. Moreover, the lower limit of the CI for micro-transactions did not approach 1.00. In fact, the lower limit was 1.94, which suggests that micro-transactions result in, at minimum, a doubling of the odds of migrating to online gambling. Taken together, we have a great deal of confidence in our findings.

## Conclusion

The gambling industry has continued to evolve and adapt to new forms of technology (Turner 2008). The recent popularity and ubiquity of the Internet has radically changed the way people engage in gambling and gambling-related activities. The gambling industry has taken full advantage of today’s wired world to offer new ways to gamble. One such example is through the help of social networking sites such as Facebook to promote their sites and attract new players. Indeed, gambling advertisements (be it social casino games or online gambling sites) are commonly found on Facebook. Whilst the current study makes an important contribution to our understanding of social casino games and online gambling, more research is needed in this important area of inquiry to minimize the potential harms of social casino gaming. Indeed, social casino games may prompt millions of people to try online gambling, which for some may result in a slippery slope towards disordered gambling. The results raise some concerns, especially for underage youth. Policy makers, the industry and clinicians would be well advised to monitor the growth of social casino gaming closely.

## Footnotes

1 We assessed whether systematic differences existed at Time 1 on our main variables of interest and revealed a gender effect. As such, we included gender as a covariate in our binary logistic model. The inclusion of gender as a covariate did not change the results of our original model.

2 To assess whether significant differences in regards to demographics (e.g., gender, age, employment and ethnicity) existed between participants who migrated to online gambling and participants who did not make the migration, we ran a series of ANOVAs and Chi-Square analyses. The results suggest that gender was the only significant difference between participants who migrated to online gambling and those who did not *χ* ^2^(2, *N* = 99) = 6.99, *p* = .01. Specifically, more male (65.38 %) than female (34.62 %) social casino gamers migrated to online gambling.
